# *Allium hookeri* root extract exerts anti-inflammatory effects by nuclear factor-κB down-regulation in lipopolysaccharide-induced RAW264.7 cells

**DOI:** 10.1186/s12906-017-1633-3

**Published:** 2017-02-23

**Authors:** Ja-Young Jang, Min-Jung Lee, Bo-Ram You, Jong-Sik Jin, Sung-Hyen Lee, Ye-Rang Yun, Hyun Ju Kim

**Affiliations:** 1 Research and Development Division, Industrial Technology Research Group, World Institute of Kimchi, Nam-Gu, Gwangju, 61755 South Korea; 2 Department of Oriental Medicine Resources, Chonbuk National University, Iksan, Jeonbuk 54596 Republic of Korea; 3 Functional Food & Nutrition Division, Department of Agro-food Resources, Rural Development Administration, Wanju, Jeonbuk 55365 South Korea

**Keywords:** *Allium hookeri*, Inflammation, iNOS, COX-2, NF-κB

## Abstract

**Background:**

*Allium hookeri* (AH) is widely consumed as a vegetable and herbal medicine in southeastern Asia. AH has been reported antioxidant, antimicrobial, improvement of bone health and antidiabetic effects. In the present study, we investigated the inhibitory effect of a methanol extract of AH root (AHE) on inflammatory response in lipopolysaccharide (LPS)-induced RAW264.7 cells.

**Methods:**

Initially, characterization of organic sulfur compounds in AHE was determined using high performance liquid chromatography-electrospray ionization-mass spectrometry (HPLC-ESI-MS). Cells were incubated with LPS and AHE for 24 h. The productions of nitric oxide (NO), reactive oxygen species (ROS), and inflammation-related cytokines were examined. Gene and protein expression of inducible nitric oxide synthase (iNOS) and cyclooxygenase-2 (COX-2) were assessed by polymerase chain reaction and Western blotting. Key factor, nuclear factor kappa B (NF-κB) was also determined.

**Results:**

AHE contained organosulfur compounds such as alliin and *S*-allylcysteine by HPLC-ESI-MS. AHE significantly inhibited NO, ROS, and cytokines production in LPS-induced RAW264.7 cells. In addition, AHE treatment inhibited iNOS and COX-2 mRNA and protein levels, leading to a decrease in iNOS-derived NO level. Furthermore, NF-κB activation was, at least in part, suppressed by AHE treatment.

**Conclusion:**

Our data suggest that AHE treatment inhibits the inflammation condition through suppression of iNOS and COX-2 expression *via* NF-κB down-regulation.

## Background

Inflammation is a biological response of the body to harmful stimuli, such as tissue damage or infection, and is an essential response to eliminate aggressors. In general, inflammation is classified with acute and chronic. Chronic inflammation is closely related to cause diseases, such as atherosclerosis, asthma, rheumatoid arthritis, inflammatory bowel disease and cancer [1]. Hence, various attempts in medicinal herb have been demonstrated for prevention and treatment of inflammation. For instance, corynoline isolated *Corydalis bungeana Turcz* showed anti-inflammatory effect by decreasing pro-inflammatory mediator including nitric oxide (NO), inducible nitric oxide synthesis (iNOS), cyclooxygenase-2 (COX-2), tumor necrosis factor-alpha (TNF-α) and interleukin-1β (IL-1β) [2]. In other study, *Viola yedoensis* also showed the anti-inflammatory effect on lipopolysaccharide (LPS)-induced RAW264.7 cells [3].

**Table 1:** Primer sequence used in RT-PCR

Genes	Forward primer (5’–3’)	Reverse primer (5’–3’)
GAPDH	CGTAGACAAATGGTGAAGGT	AATGAAGGGGTCGTTGATG
iNOS	GCCAAGCCCTCACCTACTTC	CCAGAAACTTCGGAAGGGAG
COX-2	TTCTTTGCCCAGCACTTCAC	GGTTGAAAAGGAGCTCTGGG

LPS induces the activation of monocytes and macrophages, and synthesize and release of inflammation related factors, such as TNF-α, IL-6, NO, iNOS, COX-2, and reactive oxygen species (ROS) [4]. Cytokines also produce iNOS and COX-2 as well LPS treatment. Subsequently, iNOS and COX-2 are involved in inflammation process by generation of NO and prostaglandin, respectively. While, ROS is related to activate the nuclear factor kappa B (NF-κB) by pro-inflammatory cytokines such as TNF-α [5]. The activation of NF-κB is caused by inhibitor of kB (IκB) kinases (IKKs) phosphorylation and NF-κB degradation [6]. Activated NF-κB is resultingly induce pro-inflammatory genes as a transcription factor [7, 8]. Therefore, the activation of NF-κB is a pivotal process in the inflammation of human and animal models [9]. Hence, NF-κB is a target gene to seek the anti-inflammatory compound in prevention and treatment of inflammation [10].

*Allium hookeri* Thwaites (Liliaceae family, AH) is a traditional herb in Southeast Asia. AH is introduced in 2012 and widely cultivated in South Korea. AH is mainly used as a supplementary food and medicinal food [11, 12]. AH is reported to contain higher amounts of total protein, sugar, fiber, phytosterol, ascorbic acid and total phenol with the lower amount of total fat than *A. cepa*. [11]. Surprisingly, AH root of South Korea contains twice the amount of sulfur, crude saponin, mineral content, and amino acid in comparison to Myanmar [13]. Although various biological activities of AH root and leaf have been reported, the underlying mechanisms of AH remain unclear. So far, AH root and leaf showed anti-oxidant [14–16], anti-inflammatory [17], anti-microbial [18], improvement of bone health [19], anti-obesity [20] and anti-diabetic effects [21–23]. Our previous study reported that water extract of AH leaf or root revealed anti-diabetic effects in the type 2 diabetic *db/db* mice and in the pancreatic β-cell of streptozotocin (STZ)-induced diabetic rats [23].

In previous study, we demonstrated that methanol extract of AH root (AHE) exhibited the anti-inflammatory effect LPS-induced RAW264.7 cells [24]. However, the mechanistic study was not performed. Based on these screening results, this study was to investigate the mechanism of AHE on the anti-inflammatory effect in LPS-induced RAW264.7 cells. To investigate the anti-inflammatory effect of AHE, the production of NO, ROS, and cytokine production was measured. Next, mRNA and protein levels of iNOS and COX-2 were determined. NF-κB protein level was lastly measured as a target gene.

## Methods

### Materials

(±)-L-Alliin and *S*-allylcysteine (SAC) was purchased from Sigma-Aldrich (St. Louis, MO., USA). LPS (purified from *Escherichia coli* (*E. coli*) O127: E8), penicillin-G, streptomycin, sulfanilamide, N-(1-naphtyl)ethylenediamine dihydrochloride, and 2’,7’-dichlorofluorescin diacetate (DCFH-DA) were purchased from Sigma-Aldrich (St. Louis, MO., USA). IL-6 and TNF-α Enzyme Linked Immuno Sorbent Assay (ELISA) kit was purchased from Enzo Life Science Inc. (Farmingdale, NY, USA). The primary antibodies against iNOS (#2982), COX-2 (#4842), NF-κB (p65) (#8242) and IκBα (#4812) were obtained from Cell Signaling (Cell Signaling Tech., Beverly, MA, USA). Goat anti-rabbit IgG-horseradish peroxidase (HRP) (sc-2004) and goat anti-mouse IgG-HRP (sc-2005) were obtained from Santa Cruz Biotechnology Inc. (Santa Cruz, CA, USA). Dulbecco’s modified Eagle’s medium (DMEM), fetal bovine serum (FBS), and dimethylsulfoxide (DMSO) were obtained from Hyclone Laboratories (Logan, UT, USA).

### Preparation of AHE

AH was cultivated in Sunchang-gun, Jeollabuk-do, Korea. Plant identification was done in the Sunchang Agricultural Development & Technology Center and a voucher specimen (RDAAH15) is kept in the Rural Development Administration. Fresh AH root (1 kg) was freeze-dried, powdered, and extracted with 10 volumes of methanol for 24 h at room temperature (RT). The extraction process was repeated 3 times. Then, the AHE was concentrated using a rotary evaporator and dissolved in DMSO.

### Identification of chemical components of AHE with liquid chromatography-electrospray ionization-mass spectrometry (LC/ESI/MS)

To identify the chemical components of AHE, concentrated AHE was dissolved in water into 10 mg/ml. As standard substance, alliin and SAC were dissolved in water into 0.1 mg/ml for LC/MS analysis. HPLC was performed on an Agilent 1200 system with Agilent 1200 series binary pump, and an Triple Quad LC/MS 6410 system (Agilent Technologies, Waldbronn, Germany) coupled with an ESI interface in positive ionization mode under following condition: column, TSK-gel ODS-80Ts (Tosoh Co., Tokyo, Japan 4.6 mm × 150 mm); mobile phase, water (solvent system A) and CH_3_CN (solvent system B) in a gradient mode (B from 10 to 50% in 20 min); flow rate, 0.5 ml/min, temperature, 30 °C. High-purity nitrogen was used as dry gas at a flow rate at 10 L/min, at a dry temperature of 300 °C. The ESI interface and mass spectrometric parameters were optimized to obtain maximum sensitivity.

### Cell culture

RAW264.7 cells, derived from mouse macrophages, were obtained from the American Type culture Collection (ATCC, Rockville, MD, USA). Cells were cultured in DMEM containing 10% (v/v) FBS, 2 mM L-glutamine, 100 U penicillin-G, and 100 μg/ml streptomycin at 37 °C in humidified atmosphere containing 5% CO_2_ and 95% air. When the cells were confluent state, AHE (100, 200, and 300 μg/ml) and LPS (2 μg/ml) were treated. Allyl disulfide (ADS) 1 ng/ml was used as positive control in all experiments.

### ROS assay

ROS level was measured, as follows [25]. RAW264.7 cells were co-treated with AHE (100, 200, and 300 μg/ml) and LPS (2 μg/ml) for 24 h. ADS (1 ng/ml) was used as positive control. Cells were washed with 1 mM EDTA-50 mM sodium phosphate buffer (pH 7.4), and incubated with 25 μM DCFH-DA for 30 min. The fluorescence intensity was determined at an excitation (486 nm) and emission (530 nm).

### NO assay

RAW264.7 cells were co-treated with AHE (100, 200, and 300 μg/ml) and LPS (2 μg/ml) for 24 h. NO production were indirectly determined by measuring the stable NO catabolite nitrite in medium by Griess reaction. In brief, culture medium was reacted with an equal volume of Griess reagent (1% sulfanilamide in 2.5% phosphoric acid and 0.1% N-(1-naphtyl)ethylenediamine dihydrochloride in water) for 10 min at RT. The absorbance was measured at 540 nm.

### IL-6 and TNF-α assay

The production of IL-6 and TNF-α was measured using by ELISA kit. Briefly, RAW264.7 were co-treated with AHE (100, 200, and 300 μg/ml) and LPS (2 μg/ml) for 24 h. After centrifugation, cell-free supernatants were collected for determination of IL-6 and TNF-α concentration according to the manufacturer’s protocols.

### RNA isolation and reverse transcription-PCR

Total RNA was isolated using Trizol Reagent according to the manufacturer’s instructions (Invitrogen, Life Technologies, Carlsbad, CA, USA). Total RNA (2 μg) was converted to cDNA in a series of standard 10 μl reverse transcription reactions. DNA amplification was carried out in AccuPower^Ⓡ^ PCR PreMix (Bioneer, Inc., Alameda, CA, USA). PCR was performed for 25 cycles as follows: annealing for 30s at 54 °C, extension for 1 min at 72 °C, denaturation for 30s at 94 °C. The PCR products were separated by electrophoresis on a 1% agarose gel and visualized by ethidium bromide (EtBR) staining. Glyceraldehyde-3-phosphate dehydrogenase (GAPDH) was used as loading control (Table 1).

### Western blot analysis

RAW264.7 cells were washed with PBS and lysed in an ice-cold radio immunoprecipitation buffer. Protein (50 μg) were electrophoresed in 12% sodium dodecyl sulfate-polyacrylamide electrophoresis (SDS-PAGE) gel and transferred to polyvinylidene difluoride membrane. The blocked membranes were incubated with the primary antibodies against iNOS, COX-2, NF-κB, and IκBα and subsequently incubated with second antibody. Protein density was quantitated with G BOX Chemi XT4 system (SYNGENE, Cambridge, UK). The basal levels of the proteins were normalized by analyzing the level of β-actin protein.

### Statistical analysis

All data were expressed as mean ± standard deviation (SD) of triplicate experiments. Statistical analyses were performed using GraphPad Prism 5 software (San Diego, CA, USA). One-way analysis of variance (ANOVA) was used to compare quantitative data among groups. The Bonferroni post hoc test was used if differences were found to be significant by ANOVA (*p* < 0.05).

## Results

### Identification of chemical components of AHE

**Fig. 1:**
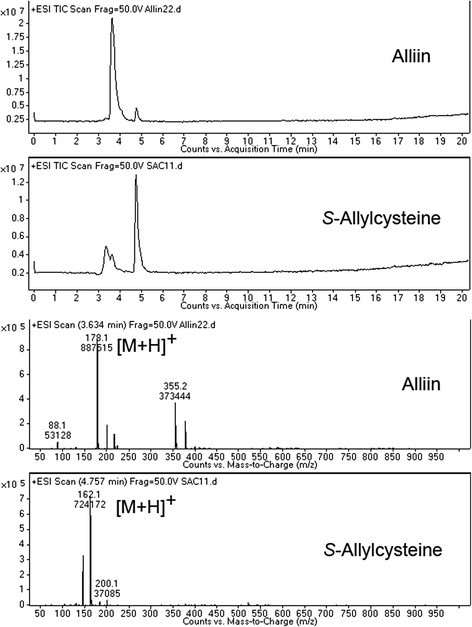
Total ion chromatogram of alliin and SAC with quasi-molecular ion peaks

**Fig. 2:**
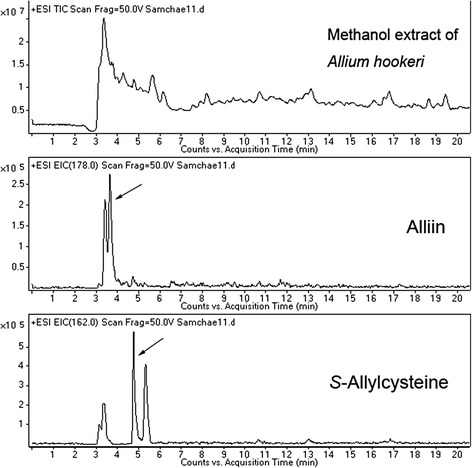
Total ion chromatogram of alliin and SAC from the methanol extract of *Allium hookeri* root (AHE)

When analyzed with standard compounds, quasi-molecular ion peaks of alliin and SAC were detected at m/z 178 [M + H]^+^ and m/z 162 [M + H]^+^, respectively (Fig. 1). The retention times of alliin and SAC were 3.6 and 4.7 min. Based on the retention time and fragmentation pattern in MS, alliin and SAC from AHE were detected (Fig. 2).

### AHE treatment inhibited NO and ROS production

**Fig. 3:**
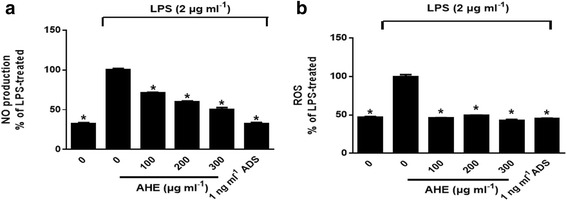
AHE decreased NO and ROS production in LPS-induced RAW264.7 cells. Cells were incubated with AHE (100, 200 and 300 μg/ml), ADS (1 ng/ml) and LPS (2 μg/ml) for 24 h. **a** NO and **b** ROS assay was performed. Data were expressed as means ± SD of triplicate tests. *p < 0.05 indicate statistically significant differences compared to LPS-treatment alone

To investigate the anti-inflammatory effects of AHE, we first examined the inhibitory effects of AHE on LPS-induced NO production in RAW264.7 cells. Extracellular (culture medium) NO levels were directly measured by quantifying its oxidized product, nitrite (NO_2_^-^). As shown in Figure 3a, a significant (*p* < 0.05) increase in NO production was observed after exposure to LPS, whereas treatment with AHE caused a sustained decrease in LPS-induced NO production in a dose-dependent manner. Next, we used a DCFH_2_-DA fluorescent staining method to determine whether AHE was able to inhibit LPS-induced NO and ROS generation in RAW264.7 cells. As shown in Figure 3b, the intracellular ROS generation was increased in LPS-treated cells as indicated by increased intensity of DCF fluorescence, whereas this LPS-induced ROS generation was significantly inhibited by AHE treatment (100 ~ 300 μg/mL).

### AHE treatment inhibited IL-6 and TNF-α production

**Fig. 4:**
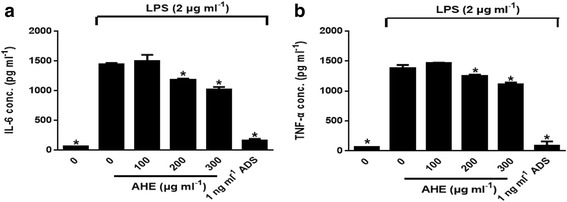
AHE decreased IL6 and TNF-α production in LPS-induced RAW264.7 cells. Cells were incubated with AHE (100, 200 and 300 μg/ml), ADS (1 ng/ml) and LPS (2 μg/ml) for 24 h. **a** IL6 and **b** TNF-α levels were measured by ELISA kit. Data were expressed as means ± SD of triplicate tests. *p < 0.05 indicate statistically significant differences compared to LPS-treatment alone

IL-6 and TNF-α production of RAW264.7 cells were essentially increased by LPS. Figure 4a showed that AHE treatment over 200 μg/ml significantly inhibited IL-6 production (*p* < 0.05). In the TNF-α production, AHE treatment inhibited by 35% in comparison to the LPS-induced control group (Fig. 4b, *p* < 0.05).

### AHE treatment inhibited iNOS and COX-2 mRNA and protein expression

**Fig. 5:**
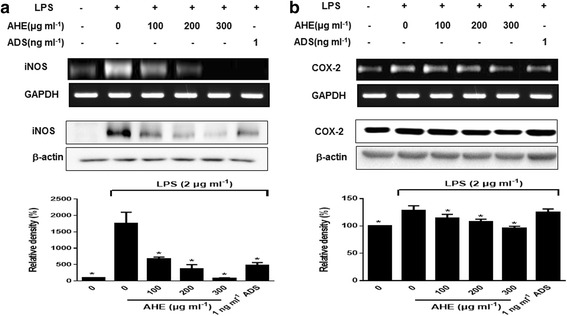
AHE inhibited the mRNA and protein level of iNOS and COX-2 in LPS-induced RAW264.7 cells. Cells were incubated with AHE (100, 200 and 300 μg/ml), ADS (1 ng/ml) and LPS (2 μg/ml) for 24 h. **a** RT-PCR and **b** western blot was performed. *p < 0.05 indicate statistically significant differences compared to LPS-treatment alone

To investigate whether AHE have an effect on mRNA and protein levels of iNOS and COX-2 or not. Treatment with LPS significantly up-regulated iNOS mRNA and protein levels of iNOS in RAW264.7 cells (Fig. 5a, *p* < 0.05). However, AHE at 300 μg/ml particularly suppressed iNOS protein level by 18-folds compared to LPS alone. In addition, COX-2 mRNA and protein level were also down-regulated by AHE treatment (Fig. 5b, *p* < 0.05).

### AHE treatment inhibits p65 NF-κB nuclear translocation

**Fig. 6:**
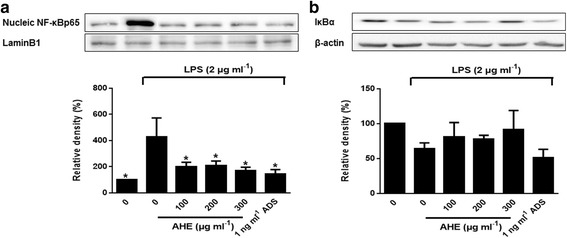
AHE suppressed p65 translocation and NF-κB and IκBα activation in LPS-induced RAW264.7 cells. Cells were incubated with AHE (100, 200 and 300 μg/ml), ADS (1 ng/ml) and LPS (2 μg/ml) for 24 h. **a** Cytosolic IκBα and **b** nuclear NF-κB were analyzed by western blot. *p < 0.05 indicate statistically significant differences compared to LPS-treatment alone

As shown in Fig. 6, nuclear NF-κB p65 was increased by LPS treatment. NF-κB translocation is reduced by AHE in RAW264.7 cells compared to LPS alone (*p* < 0.05). However, AHE treatment increased cytosolic IκBα protein level, but this increase was not significant compared to the control. These results suggested that the AHE treatment partially affected NF-κB activation in response to LPS-induction through phosphorylation of NF-κB.

## Discussion

AH is a wild herb cultivated in India, Myanmar, Nepal, and China. AH is mainly used as food supplement and medicinal food. Since, AH is introduced in South Korea, the beneficial effects of AH has been extensively investigated including, anti-oxidant [14–16], anti-inflammatory [17], anti-microbial [18], anti-obesity [20] and anti-diabetic [21–23]. Its beneficial effect is attributed to the sulfur compounds, phenolic compounds, phytosterols and vitamin C of AH [11]. Allicin and ten alkyl thiosulfinates from AH root extract were characterized by HPLC-ESI-MS [26]. Non-volatile organosulfur compounds, methiin and cycloalliin were detected as major compounds and volatile compounds such as allyl methyl sulphides and dimethyl sulphides were identified from AH [27]. Some of chemicals in AH have showed biological activities. Ferulic acid-4-*O*-β-D-glucopyranoside isolated from AH root has antioxidant activity [14]. The hot-water extract of AH containing alliin, sinapic acid, and ferulic acid exhibited beneficial effects on bone health [19].

We previously demonstrated that water extracts of AH root ameliorated oxidative stress-induced inflammatory responses and β-cell damage in pancreas of STZ-induced diabetic rats [23]. In addition, supplement of AH root lowered blood glucose and increased insulin immunoreactives cells in the type 2 diabetic *db/db* mice [21]. In this study, we found that AHE effectively suppressed LPS-induced inflammation. AHE treatment inhibited increased NO, ROS, proinflammatory cytokines in RAW264.7 cells stimulated with LPS. AHE also significantly decreased the expression of iNOS and COX-2 through inhibiting NF-κB activation.

Macrophage activation by LPS, a component of the outer membrane of Gram-negative bacteria, promotes the synthesis and release of large amounts of mediators involved in the inflammatory onset such as cytokines, NO, pro-inflammatory enzymes and ROS [28]. Accumulating evidence has indicated that NO is well known for its involvement in the development of inflammation. NO has important functions as signaling molecules in diverse physiological systems, such as cardiovascular, nervous and immunological systems [29]. High concentration of NO synthesized by iNOS, can mediate inflammation and cause cell death by inducing apoptosis [30]. Therefore, identifying new agents capable of lowering the production of this proinflammatory agent is regarded as an essential requirement for the alleviation of a number of inflammation-related disorders attributed to macrophage activation [10]. ROS have also been reported to be involved in the activation of NF-κB by pro-inflammatory cytokines such as TNF-α [31]. NF-κB has been reported to play a pivotal role in inflammatory response through the induction of inflammation-related cytokines (i.e. IL-6, IL-1β, TNF-α) and enzymes such as COX-2 and iNOS [32, 33]. In this study, we demonstrate that AHE treatment significantly inhibited NO production and iNOS expression at a concentration 100–300 μg/mL in a dose-dependent manner in RAW264.7 cells. In particular, the profound inhibition of LPS-induced NO and ROS was observed at a concentration of 100 μg/mL of AHE. In addition, our previous study also demonstrated that water extract of AH root (100 mg/kg body weight) significantly inhibited ROS production and protein expression of cytokines in the pancreas of STZ-induced diabetic rats [23].

In this study, the anti-inflammatory effect of AHE may be mediated by its inhibitory effect on the pro-inflammatory cytokines, including IL-6 and TNF-α, as well as the hallmarks of inflammation, NO, and activation of NF-κB at least in part. NF-κB is composed mainly of two proteins, p50 and p65. In resting cells, the NF-κB heterodimer is held in the cytosol through interaction with IκB inhibitory proteins [34, 35]. According to the pro-inflammatory stimuli, IκB becomes phosphorylated, ubiquitinated, and then degraded. Thus, the liberated NF-κB dimers are translocated to nucleus, where the transcription of target gene is induced [10]. We observed that AHE decreased the LPS-induced nuclear accumulation of the p65 subunit of NF-kB, but not that of IκBα, in LPS-activated RAW264.7 macrophages. Other studies have showed that the inhibitory effects of sulfur-containing compounds from garlic towards LPS-activated NF-κB dependent pathway [36]. The major water-soluble sulfur compound, SAC, in garlic extract seems to have direct inhibitory effect on NF-κB and indirect inhibitory effect on LPS-induced IL-1 and TNF-α in human whole blood [36]. The lipid-soluble sulfur compounds allicin and diallyl disulfide (DADS) also inhibit NF-κB and reduce the expression of iNOS in LPS-stimulated macrophages [36]. Dially trisulfide (DATS), an organic polysulfide compound found in garlic, attenuates the initiation of LPS-mediated intracellular signaling cascades by suppressing activation of NF-κB and by inhibiting binding of LPS to toll like receptor4 on macrophages [37]. DADS also reduced the airway inflammation *via* regulation of nuclear factor E2-related factor 2/heme oxygenase (HO)-1 and NF-κB [38]. Accumulated data demonstrated that the NF-κB pathway mediates the expression of iNOS, COX-2 and various pro-inflammatory cytokines [35]. DATS effectively suppressed phosphorylation and the degradation of IκB in RAW264.7 cells, and downregulated serine/threonine protein kinase B/transforming growth factors-β-activated kinase-mediated mitogen-activated protein kinase (MAPK) and NF-κB signaling pathways [39]. Several lines of study have demonstrated that DADS suppressed LPS-induced MAPKs signaling to attenuate inflammation responses [40]. Ethanol extracts of AH root exerts anti-inflammatory through up-regulation of HO-1 and deactivation of p38 in the LPS-stimulated RAW264.7 cells [17]. In agreement with our study, Kim et al. reported similar results. The anti-inflammatory activities of aged black garlic water extract were similar to those of raw garlic extract at nontoxic concentrations up to 250 μg/ml. Both garlic extracts inhibited cytokine production and activation of MAPK and NF-κB signal transduction pathway induced by LPS stimulated RAW264.7 cells and LPS-induced lethal shock in mice [41]. The findings from our study reveal, for the first time, a protective effect of AHE in the LPS-induced RAW264.7 cells. This effect, in part, may have been contributed by the anti-inflammatory effects of sulfur compounds in AHE. Therefore, we believe that AHE could potentially be used in the treatment of inflammation related diseases.

## Conclusions

In conclusion, the present study demonstrated the anti-inflammatory effects of AHE in LPS-induced RAW264.7 cells. AHE treatment inhibited NO and ROS production and it reduced proinflammatory mediators, which was accompanied by iNOS and COX-2 expression *via* downregulating NF-kB signaling pathways in RAW264.7 cells stimulated by LPS. These results suggest that AHE may be useful for the prevention of various diseases associated with inflammation. Therefore, our future studies will focus on providing additional mechanical evidence to demonstrate this possibility in vivo.

## Abbreviations

ADS: Allyl disulfide
AH: *Allium hookeri*
AHE: Methanol extract of AH root
AKT: Serine/threonine protein kinase B
COX-2: Cyclooxygenase-2
DADS: Diallyl disulfide
DATS: Diallyl trisulfide
HO-1: Heme oxygenase-1
IL-1β: Interleukin-1β
iNOS: Inducible nitric oxide synthase
IκB: Inhibitor of kB
LC-ESI-MS: Liquid chromatography-electrospray ionization-mass spectrometry
LPS: Lipopolysaccharide
NF-κB: Nuclear factor kappa B
NO: Nitric oxide
Nrf2: Nuclear factor E2-related factor 2
ROS: Reactive oxygen species
SAC: *S*-allycysteine
STZ: Streptozotocin
TNF-α: Tumor necrosis factor-alpha
