# Erratum to: *Allium hookeri* root extract exerts anti-inflammatory effects by nuclear factor-κB down-regulation in lipopolysaccharide-induced RAW264.7 cells

**DOI:** 10.1186/s12906-017-1709-0

**Published:** 2017-03-28

**Authors:** Ja-Young Jang, Min-Jung Lee, Bo-Ram You, Jong-Sik Jin, Sung-Hyen Lee, Ye-Rang Yun, Hyun Ju Kim

**Affiliations:** 1 Research and Development Division, Industrial Technology Research Group, World Institute of Kimchi, Nam-Gu, Gwangju, 61755 South Korea; 2 Department of Oriental Medicine Resources, Chonbuk National University, Iksan, Jeonbuk 54596 Republic of Korea; 3 Functional Food & Nutrition Division, Department of Agro-food Resources, Rural Development Administration, Wanju, Jeonbuk 55365 South Korea

## Erratum

Upon publication of the original article [1], the grant number referenced in this paper was KE1701-3. This was incorrect and has since been changed to reflect the correct grant reference of KE1301-1.
